# Financial Hardship Among Pregnant and Postpartum Women in the United States, 2013 to 2018

**DOI:** 10.1001/jamanetworkopen.2021.32103

**Published:** 2021-10-29

**Authors:** Kathryn Taylor, Sarah Compton, Giselle E. Kolenic, John Scott, Nora Becker, Vanessa K. Dalton, Michelle H. Moniz

**Affiliations:** 1 National Clinician Scholars Program, University of Michigan, Ann Arbor; 2 Department of General Surgery, Stanford University, Stanford, California; 3 Department of Obstetrics and Gynecology, University of Michigan, Ann Arbor; 4 Department of Surgery, University of Michigan, Ann Arbor; 5 Institute for Healthcare Policy and Innovation, University of Michigan, Ann Arbor; 6 Division of General Medicine, Department of Internal Medicine, University of Michigan, Ann Arbor; 7 Program on Women’s Healthcare Effectiveness Research (PWHER), University of Michigan, Ann Arbor

## Abstract

**Importance:**

Financial hardship affects health care access and health outcomes among peripartum women.

**Objective:**

To evaluate the prevalence of financial hardship among peripartum women over time and by insurance type and income.

**Design, Setting, and Participants:**

This cross-sectional study included peripartum women, defined as women aged 18 to 45 years who reported being currently pregnant or pregnant in the past 12 months, who participated in the National Health Interview Survey from 2013 to 2018. Data were analyzed from January to May 2021.

**Exposures:**

Current pregnancy or recent pregnancy as well as insurance type and income.

**Main Outcomes and Measures:**

Three measures of financial hardship within the last year were evaluated: (1) unmet health care need due to cost (unmet need for medical care or delayed or deferred medical care due to cost); (2) health care unaffordability (worry about paying for potential medical bills or existing medical debt); and (3) general financial stress (worry about subsistence spending [eg, monthly bills, housing]).

**Results:**

The study cohort included 3509 peripartum women, weighted to represent 1 050 789 women (2018: an estimated 36 045 of 184 018 [19.6%] Hispanic, 39 017 [21.2%] Black, and 97 366 [52.9%] White), with a mean (SD) age of 29 (6) years. Overall, from 2013 to 2018, 24.2% (95% CI, 22.6%-26.0%) of peripartum women reported unmet health care need, 60.0% (95% CI, 58.0%-61.9%) reported health care unaffordability, and 54.0% (95% CI, 51.5%-56.5%) reported general financial stress. The prevalence of financial hardship outcomes did not substantially change between 2013 and 2018 (unmet health care need in 2013: 27.9% [95% CI, 24.4%-31.7%]; in 2018: 23.7% [95% CI, 19.5%-28.6%]; health care unaffordability in 2013: 65.7% [95% CI, 61.1%-70.0%]; in 2018: 58.8% [95% CI, 53.4%-64.0%]; general financial stress in 2013: 60.6% [95% CI, 55.2%-65.8%]; in 2018: 53.8% [95% CI, 47.8%-59.8%]). Women with private insurance had lower odds of unmet need (adjusted odds ratio [aOR], 0.67; 95% CI, 0.52-0.87) but higher odds of health care unaffordability (aOR, 1.88; 95% CI, 1.49-2.36) compared with women with public insurance. Peripartum women with household incomes less than 400% of the federal poverty level had higher odds of unmet need (aOR, 1.50; 95% CI, 1.08-2.08) and unaffordable care (aOR, 1.98; 95% CI, 1.54-2.55) compared with those with household incomes 400% or more of federal poverty level.

**Conclusions and Relevance:**

These findings suggest that financial hardship among peripartum women in the United States was common from 2013 to 2018, including 24% of pregnant and postpartum women reporting unmet health care need and 60% reporting health care unaffordability. Women with private insurance and those living on lower incomes were more likely to experience unaffordable health care than women with pubic insurance and those with higher incomes, respectively. Targeted policy interventions are needed to improve health care affordability and promote overall economic security among peripartum women.

## Key Points

**Question:**

What is the prevalence of financial hardship, including unmet health care need due to cost, health care unaffordability, and general financial stress, among peripartum women in the United States?

**Findings:**

This cross-sectional study of 3509 peripartum women, weighted to represent more than 1 million women, found that financial hardship was common: from 2013 to 2018, 24% reported unmet health care needs; 60%, health care unaffordability; and 54%, general financial stress. Private insurance was associated with lower odds of unmet health care need but higher odds of health care unaffordability, and lower household income was associated with higher odds of both unmet health care need and health care unaffordability.

**Meaning:**

These findings suggest that financial hardship is highly prevalent among peripartum women, which should prompt policy interventions to promote the economic well-being of families.

## Teaser

This cross-sectional study evaluates the prevalence of financial hardship, including as unmet health care need due to cost, health care unaffordability, and general financial stress, among peripartum women over time and by insurance type and income.

## Introduction

The peripartum period, defined as pregnancy and the 12 months after delivery, is a critical time for health care access for women and infants.^1^ Prenatal and postpartum visits provide essential preventive services (eg, vaccinations, screening for gestational diabetes and anemia) and an opportunity for early diagnosis and management of pregnancy complications. However, the cost of health care is a well-recognized barrier to utilization of recommended services^2,3^ and can affect both those without insurance (who are exposed to full charges for health care services^4^) and those with insurance (via copayments, coinsurance, and deductibles^5^). Those who delay or forego medical care due to financial hardship are more likely to report worse health.^6^ In addition to delayed care, financial hardship has also been shown to be associated with poor mental health.^7,8,9^ Improving coverage and affordability of recommended health care for peripartum women is a decades-long policy goal, beginning with the Pregnancy Discrimination Act in the 1970s and the expansion of Medicaid eligibility for pregnant and postpartum women in the 1980s. More recently, the Patient Protection and Affordable Care Act (ACA) included a preventive services provision that requires coverage for some prenatal services without cost-sharing for those with private insurance, and multiple other provisions sought to broadly expand coverage and affordability for all women of reproductive age. These policies appear to be benefitting women overall, with improved insurance coverage and reduced cost-related barriers to care—especially among those living on low incomes.^10,11^

However, it is unclear whether these broader trends reflect the experience of peripartum women, or rather, if additional policy change is needed to ensure the financial stability of this group. In a 2020 study of peripartum women with private insurance, out-of-pocket costs for maternity care increased by nearly 50% between 2008 and 2015, largely driven by increasing deductible payments.^12^ Additionally, many families with private insurance are facing increasing premium costs.^13^ It is unclear how these broader trends in health insurance benefit design are affecting peripartum women’s economic well-being. Moreover, there is increasing recognition that efforts to mitigate financial hardship among peripartum women may have too narrowly focused on the costs of health care itself, with relative underattention to financial hardship related to the social determinants of health, such as housing and food insecurity.^14,15,16^ Our objective in this study was to evaluate the prevalence of financial hardship among peripartum women, over time and by insurance type and household income.

## Methods

This cross-sectional, observational study used data from the 2013 to 2018 National Health Interview Survey (NHIS) to evaluate prevalence and changes over time in financial hardship among pregnant women. This study using publicly available, deidentified data was deemed not regulated by the University of Michigan institutional review board; therefore, informed consent was not required. This study followed the Strengthening the Reporting of Observational Studies in Epidemiology (STROBE) reporting guideline.

### Data Source and Study Sample

The NHIS is an annual nationally representative survey of civilian households conducted by the US Centers for Disease Control and Prevention (CDC), which collects information about demographic characteristics, socioeconomic status, health insurance, and health care access and utilization. Survey outcomes are based on a 12-month recall period. The study cohort included peripartum women, defined as women aged 18 to 45 years who reported being currently or recently pregnant (within the last calendar year). We examined data for all financial hardship outcomes beginning in 2013 (the first year for which these outcomes became available) through 2018 (the most recent year of reporting).

### Outcomes

Primary outcomes included 3 measures of financial hardship: unmet health care need due to cost, health care unaffordability, and general financial stress. *Unmet health care need due to cost* was defined as reporting needing but being unable to afford medical care, prescription medications, eyeglasses, or mental health care due to cost or reporting that the respondent or family member delayed or deferred needed care due to cost. *Health care unaffordability* was defined as reporting feeling “somewhat worried” or “very worried” about potential medical bills or reports of existing medical debt, ie, problems paying medical bills, medical bills being paid off over time, or being unable to pay medical bills. *General financial stress* was defined as reporting feeling “moderately worried” or “very worried” about monthly bills, housing costs, minimum payments on credit cards, retirement contributions, or maintaining standard of living. See eMethods in the Supplement for the detailed survey questions used for each of these outcomes.

### Statistical Analysis

We compared changes in the survey-weighted proportions of study participants’ demographic and socioeconomic characteristics as well as insurance type from 2013 to 2018 using χ^2^ tests. We evaluated the unadjusted survey-weighted proportion of each outcome overall during 2013 to 2018 to understand overall prevalence of each financial hardship outcome.

We also examined trends in prevalence over time using the unadjusted survey-weighted proportion of each financial hardship outcome in each study year. Unadjusted logistic regression was used to assess for significant variation between 2013 and 2018.

Finally, we performed stratified analyses by insurance type and income. Subpopulation analyses assessed trends among peripartum women by insurance types or income over time, with insurance types defined as private, public (Medicaid) or other, and uninsured. To isolate the association of insurance type and income with financial hardship, we used multivariable logistic regression models to estimate odds ratios (ORs) and estimated probabilities. Models were adjusted for factors that could affect the risk of financial hardship, including age, race, income-to–federal poverty level (FPL) ratio, education, marital status, employment status, insurance type, region, family size, reported health status, hospitalization in the past year, and year of survey. Race and ethnicity were reported by survey respondents and recoded by the NHIS to Hispanic, non-Hispanic Asian, non-Hispanic Black, non-Hispanic White, and non-Hispanic all other race groups. We included the following categories: Hispanic; non-Hispanic Black; non-Hispanic White; and non-Hispanic other race, which combined non-Hispanic Asian and non-Hispanic all other race groups.

All analyses used the survey weights and setup variables as provided by the NHIS to accommodate the complex sample design. Individuals missing covariates were excluded from the regression analysis but included in the overall prevalence estimates as long as they reported the primary outcome. All analyses were conducted with a 95% CI in Stata version 16 (StataCorp). Statistical significance was set at *P* < .05, and all tests were 2-tailed.

## Results

The study population included a total of 3509 peripartum women (weighted to represent 1 050 789 women) from 2013 to 2018, with a mean (SD) age of 29 (6) years. In 2018, an estimated 39 017 of 184 018 (21.2%) were Black; 36 045 (19.6%), Hispanic; and 97 366 (52.9%), White. In later study years, peripartum women were older, more highly educated, and less likely to be uninsured (Table).

**Table.** Peripartum Women, Demographic and Socioeconomic Characteristics

Characteristic	Women, Weighted No. (%)		*P* value
	2013	2018	
Weighted No. (No.)	186 543 (729)	184 018 (419)	NA
Age, y			
18-24	58 701 (31.5)	37 315 (20.3)	.003
25-34	97 999 (52.5)	115 293 (62.7)	
35-45	29 842 (16.0)	31 411 (17.1)	
Race and ethnicity			
Hispanic	38 831 (20.8)	36 045 (19.6)	.09
Non-Hispanic Black	35 753 (19.2)	39 017 (21.2)	
Non-Hispanic White	97 397 (52.2)	97 366 (52.9)	
Non-Hispanic, other race	14 562 (7.8)	11 590 (6.3)	
Tax unit income			
<400% of FPL	134 165 (76.3)	120 903 (67.6)	
≥400% of FPL	41 789 (23.8)	57 874 (32.4)	.10
Insurance			
Private	83 319 (44.9)	94 626 (51.4)	
Public or other	75 850 (40.9)	72 273 (39.3)	.04
Uninsured	26 425 (14.2)	17 118 (9.3)	
Marital status			
Married	102 841 (55.1)	113 403 (61.6)	
Lives with partner	23 627 (12.7)	25 731 (14.0)	.11
Widowed, separated, or divorced	12 649 (6.8)	5674 (3.1)	
Never married	47 426 (25.4)	39 211 (21.3)	
Family size, mean (SD)	3 (2)	4 (1)	
Education			.14
Less than high school	26 433 (14.2)	24 050 (13.2)	
High school diploma	82 519 (44.4)	73 998 (40.5)	.01
Some college	60 493 (32.5)	56 252 (30.8)	
College degree	16 539 (8.9)	28 377 (15.5)	
Employment status			
Employed	83 443 (44.7)	95 801 (52.1)	
Unemployed	103 099 (55.3)	88 217 (47.9)	.07
Self-reported health status			
Excellent or very good	134 839 (72.3)	135 425 (73.6)	
Fair or poor	12 105 (6.5)	7702 (4.2)	.38
Hospitalized in past year	73 957 (39.7)	73 402 (39.9)	.50
Region			.50
Northeast	27 660 (14.8)	30 996 (16.8)	
Midwest	42 911 (23.0)	39 637 (21.5)	.80
South	76 575 (41.1)	73 636 (40.0)	
West	39 397 (21.1)	39 750 (21.6)	
		

Abbreviations: FPL, federal poverty level; NA, not applicable.

### Overall Prevalence of and Trends in Financial Hardship, 2013 to 2018

**Figure 1.**
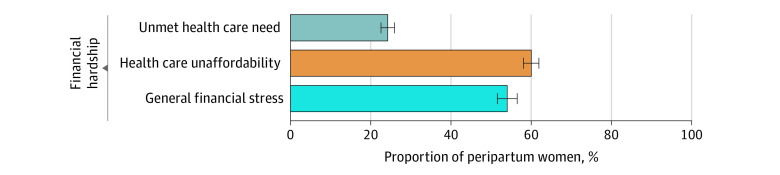
Financial Hardship Among Peripartum Women From 2013 to 2018 This figure represents unadjusted, survey-weighted proportions of each outcome reported by peripartum women from the combined years of 2013-2018. Error bars show 95% CIs.

**Figure 2.**
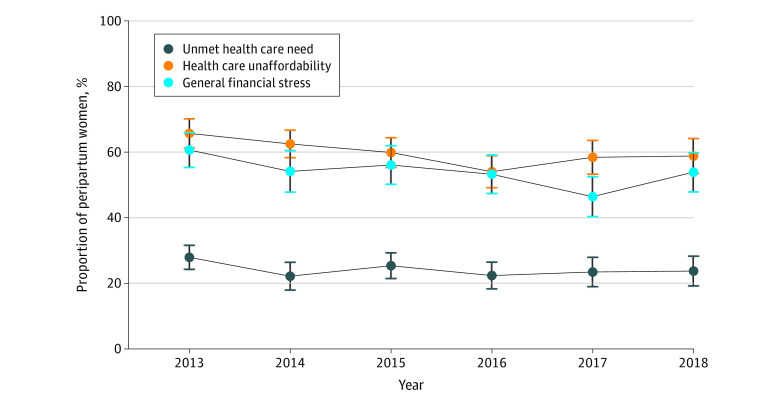
Trends in Financial Hardship Outcomes Among Peripartum Women From 2013 to 2018 This figure represents unadjusted, survey-weighted proportions of each outcome reported by peripartum women by year. Error bars show 95% CIs.

Overall, from 2013 to 2018, 24.2% (95% CI, 22.6%-26.0%) of peripartum women reported unmet health care need, 60.0% (95% CI, 58.0%-61.9%) reported health care unaffordability, and 54.0% (95% CI, 51.5%-56.5%) reported general financial stress (Figure 1). Comparing the unadjusted reported financial hardship outcome by each study year, unmet health care need (2013: 27.9% [95% CI, 24.4%-31.7%]; 2018: 23.7% [95% CI, 19.5%-28.6%]), health care unaffordability (2013: 65.7% [95% CI, 61.1%-70.0%]; 2018: 58.8% [95% CI, 53.4%-64.0%]), and general financial stress (2013: 60.6% [95% CI, 55.2%-65.8%]; 2018: 53.8% [95% CI, 47.8%-59.8%]) did not change substantively between 2013 and 2018 (Figure 2).

### Stratified Analysis of Financial Hardship by Insurance Type and Income

**Figure 3.**
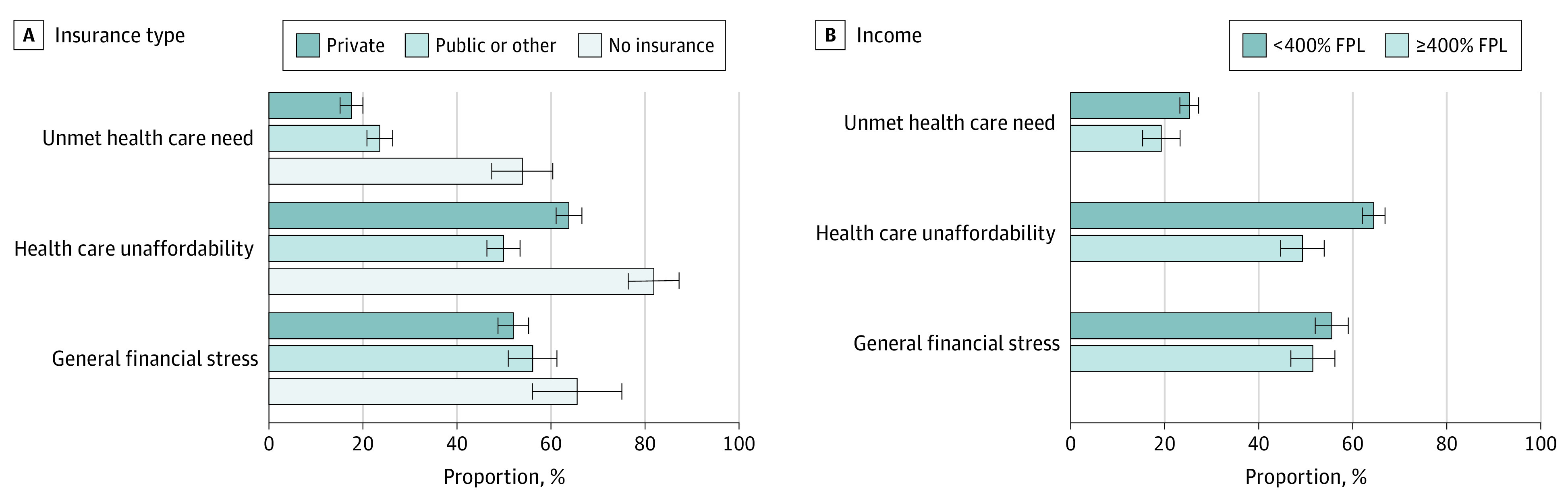
Estimated Probabilities of Financial Hardship Among Peripartum Women by Insurance Type and Income This figure represents estimated probabilities of each financial hardship outcome for combined study years 2013 to 2018, adjusted for age, race, income-to–federal poverty level (FPL) ratio, education, marital status, employment status, insurance type, region, family size, reported health status, hospitalization in the past year, and year of survey. Error bars show 95% CIs.

All financial hardship outcomes were common across all insurance and income groups (eg, unaffordable care with private insurance: 63.8% [95% CI, 61.1%-66.6%]; with public insurance: 49.9% [95% CI, 46.4%-53.4%]; with no insurance: 81.8% [95% CI, 76.4%-87.3%]; with income <400% of the FPL: 65.5% [95% CI, 62.1%-66.9%]; with income ≥400% of the FPL: 49.3% [95% CI, 44.7%-53.9%]) (Figure 3). Uninsured peripartum women had the highest odds of reporting unmet health care need (adjusted OR [aOR], 4.40; 95% CI, 3.23-6.00) and health care unaffordability (aOR, 5.18; 95% CI, 3.49-7.70) compared with women with public insurance, while women with private insurance had lower odds of reporting unmet health care need (aOR, 0.67; 95% CI, 0.52-0.87) but higher odds of reporting health care unaffordability (aOR, 1.88; 95% CI, 1.49-2.36) compared with women with public insurance (eTable in the Supplement). Peripartum women with household incomes of less than 400% of the FPL had higher odds of reporting unmet health care need (aOR, 1.50; 95% CI, 1.08-2.08) and health care unaffordability (aOR, 1.98; 95% CI, 1.54-2.55) compared with those with household incomes of at least 400% of FPL. Odds of general financial stress did not differ by insurance status or income.

## Discussion

This cohort study using nationally representative survey data found persistently high prevalence of financial hardship among peripartum women between 2013 and 2018. Despite the implementation of the ACA and a generally strong economy during the study period, financial hardship did not improve. Compared with women with public insurance, those without insurance were more likely to experience unmet health care need and health care unaffordability and those with private insurance were less likely to report unmet health care need but more likely to report health care unaffordability. General financial stress was highly prevalent, affecting at least 5 in 10 peripartum women in all income and insurance groups. Policies targeting financial risk protection are urgently needed to promote improved economic well-being of peripartum women and their growing families.

To our knowledge, this is the first study to quantify broader financial hardship among peripartum women in the United States beyond medical costs alone. General financial stress, including worry about paying monthly bills, credit cards, and housing expenses, was common among peripartum women. This financial hardship may reflect economic burden due to health care as well as a host of other circumstances contributing to financial instability.^17^ Household income falls dramatically around the time of childbirth,^18^ with the United States as the only resourced country without mandatory paid parental leave.^19^ Rent costs are rising faster than renters’ wages, putting many families at risk of eviction—more than 2 million families were evicted in 2016, with low-income, Black, and Hispanic families particularly at risk.^20,21,22^ Policies and programs that address social needs during pregnancy have clear benefits for birth and developmental outcomes, such as preterm birth and low birth weight,^14,15,23,24,25,26,27^ and may help to alleviate the highly prevalent general financial stress observed in this study.

Our findings also underscore the importance of stable insurance coverage for pregnant and postpartum women. At all study time points, rates of unmet health care need and health care unaffordability were highest among peripartum women without health insurance coverage. These financial burdens may help to explain why lack of insurance during pregnancy is associated with inadequate and late prenatal care and elevated risk of adverse birth outcomes.^4,28^ Disruptions in insurance coverage are common during pregnancy and the first 6 months post partum, affecting women with both private and public insurance.^29,30^ Specifically, individuals insured by Medicaid at time of childbirth may lose insurance coverage given the change in eligibility requirements after delivery.^29^ Expansion of peripartum insurance coverage is a proven strategy to improve access to care and maternal and infant health outcomes.^26,31,32^ In addition, Medicaid expansion has been associated with decreased housing evictions.^33^ Our findings suggest that improved coverage may also alleviate the financial hardship currently faced by many families in the United States.

However, financial hardship was extremely common among peripartum women regardless of insurance. Health care unaffordability was pervasive, affecting more than 50% of women with insurance and a striking 64% of women with private insurance. Our results suggest that perinatal health care may be leading families to be worried about paying medical bills or experiencing medical debt. This study also newly documents high rates of unmet health care need among peripartum women with insurance (18% of women with private insurance and 24% of those with public insurance), suggesting that financial hardship may be leading some peripartum women to delay or defer recommended health care services. For women with private insurance, these findings may reflect high out-of-pocket costs for pregnancy care and for maternal and infant care during childbirth.^12,34,35^ For women insured by Medicaid, who are largely shielded from out-of-pocket costs for pregnancy care, the observed financial hardship may reflect the indirect costs associated with health care, such as those for childcare, transportation, parking, and unpaid time off work related to health care use. While improving health coverage and benefit design is an important policy priority, our results demonstrate that these policy changes alone will likely be insufficient. Alleviating unaffordable care or unmet health care need for peripartum individuals will likely require intersectional changes, such as paid parental leave, child tax credits, and strategies to alleviate food and housing insecurity.

Alleviating health care–related financial hardship among peripartum women is a long-standing, bipartisan policy goal. Our findings suggest urgent need for policies that ensure uninterrupted insurance coverage for pregnant and postpartum women. Extension of Medicaid coverage from 60 days to a full year post partum is one such policy, which is supported by several federal legislative proposals, including the Helping Medicaid Offer Maternity Services (MOMS) Act of 2019 and the Patient Protection and Affordable Care Act Enhancement Act.^36^ Multiple states are also pursuing actions to extend postpartum Medicaid eligibility.^36,37,38^ Policy makers should also consider policies to reduce or eliminate cost-sharing for recommended health care during pregnancy and the year post partum as a means to improve families’ financial stability and ensure utilization of recommend maternal and infant health care services. Our findings related to highly prevalent general financial stress also call for policies to address unmet social needs during pregnancy and the postpartum year (eg, state or federal policies preventing eviction during this time could reduce risk for adverse birth outcomes and improve families’ economic resilience) as well as paid parental leave.^19^

### Limitations

This study must be interpreted in light of its limitations. First, the NHIS is a survey database and contains little clinical information. The study population and outcomes are defined by participant responses. However, these are ideally suited to our evaluation of the prevalence of financial hardship. Second, the cross-sectional survey design limits longitudinal individual-level comparisons. Therefore, we are only able to examine the peripartum period cross-sectionally and cannot evaluate dynamic changes in financial hardship at the individual level (ie, before vs after delivery). In addition, while the survey is designed to be nationally representative, the absolute numbers of peripartum women included in the survey limit some smaller subgroup analyses. Additionally, this study focused on pregnant and postpartum women, and the results are not generalizable to all women.

## Conclusions

In this study representing more than 1 million peripartum women, unmet health care need, health care unaffordability, and general financial stress were common. Despite the study period including the implementation of the ACA and a generally strong economy, financial hardship remained prevalent among this population. While private insurance was associated with greater health care unaffordability, respondents without insurance were at the highest risk of all outcomes. As the future of the ACA and Medicaid expansion continues to be debated, ensuring continuous insurance for the peripartum population and reducing cost-sharing to improve access to essential perinatal services are critical.

## Notes

Supplement. **eMethods. **Survey Questions for Outcomes **eTable. **Adjusted Odds of Financial Hardship Among Peripartum Women, 2013-2018
