# Supplementary material for: Financial Hardship Among Pregnant and Postpartum Women in the United States, 2013 to 2018
Source: JAMA Netw Open. 2021 Oct 29;4(10):e2132103. doi: 10.1001/jamanetworkopen.2021.32103 (PMC8556621; doi:10.1001/jamanetworkopen.2021.32103)
Supplement: Supplement. — eMethods. Survey Questions for Outcomes eTable. Adjusted Odds of Financial Hardship Among Peripartum Women, 2013-2018 [file jamanetwopen-e2132103-s001.pdf]

Supplemental Online Content

Taylor K, Compton S, Kolenic GE, et al. Financial hardship among pregnant and postpartum women in the United States, 2013 to 2018. *JAMA Netw Open.* 2021;4(10):e2132103. doi:10.1001/jamanetworkopen.2021.32103

eMethods. Survey Questions for Outcomes

eTable. Adjusted Odds of Financial Hardship Among Peripartum Women, 2013-2018

This supplemental material has been provided by the authors to give readers additional information about their work.

© 2021 Taylor K et al. *JAMA Network Open.*

eMethods. Survey Questions for Outcomes:

If a respondent answered yes to any of following survey questions, they were considered to have the corresponding outcome.

1. Unmet Healthcare Need
    a. Unmet Need for Medical Care
    b. Delayed or Deferred Care
2. Healthcare Unaffordability
    a. Worry About Medical Bills
    b. Medical Debt
3. General Financial Stress
    a. Normal Bills
    b. Housing
    c. Credit Cards
    d. Retirement
    e. Maintaining Standard of Living

1. Unmet Healthcare Need
    a. Unmet Need for Medical Care
        i. *During the past 12 months, was there any time when you needed any of the following, but didn't get it because you couldn't afford it?*
            1. Prescription medicines
            2. Mental health care or counseling
            3. Dental care (including check ups)
            4. Eyeglasses
    b. Delayed or Deferred Care
        i. [have you delayed seeking medical care/has medical care been delayed for anyone in the family] because of worry about the cost?
        ii. was there any time when [fill1: you/someone in the family] needed medical care, but did not get it because [fill2: you/the family] couldn't afford it?
2. Healthcare Unaffordability
    a. Worry About Medical Bills
        i. If you get sick or have an accident, how worried are you that you will be able to pay your medical bills? Are you…
            1. Very worried
            2. Somewhat worried
            3. Not at all worried
    b. Medical Debt
        i. In the past 12 months did [you/anyone in the family] have problems paying or were unable to pay any medical bills? Include bills for doctors, dentists, hospitals, therapists, medication, equipment, nursing home or home care.
        ii. [Do you/Does anyone in your family] currently have any medical bills that you are unable to pay at all?
        iii. [Do you/Does anyone in your family] currently have any medical bills that are being paid off over time? This could include medical bills being paid off with a credit card, through personal loans, or bill paying arrangements with hospitals or other providers. The bills can be from earlier years as well as this year.
3. General Financial Stress
    a. Normal Monthly Bills
        i. How worried are you right now about not having enough to pay your normal monthly bills? Are you…
            1. Very worried
            2. Moderately worried
            3. Not too worried
            4. Not worried at all
    b. Housing

© 2021 Taylor K et al. *JAMA Network Open.*

i. How worried are you right now about not being able to pay your rent, mortgage, or other housing costs? Are you…
    1. Very worried
    2. Moderately worried
    3. Not too worried
    4. Not worried at all

c. Credit Cards
    i. How worried are you right now about not being able to make the minimum payments on your credit cards? Are you…
        1. Very worried
        2. Moderately worried
        3. Not too worried
        4. Not worried at all

d. Retirement
    i. How worried are you right now about not having enough money for retirement? Are you…
        1. Very worried
        2. Moderately worried
        3. Not too worried
        4. Not worried at all

e. Maintaining Standard of Living
    i. How worried are you right now about not being able to maintain the standard of living you enjoy? Are you…
        1. Very worried
        2. Moderately worried
        3. Not too worried
        4. Not worried at all

© 2021 Taylor K et al. *JAMA Network Open.*

eTable. Adjusted Odds of Financial Hardship Among Peripartum Women, 2013-2018

Covariable	Unmet Healthcare Need	Healthcare Unaffordability	General Financial Stress
Age			
18-24	ref	ref	ref
25-34	1.01 (0.79-1.31)	1.62 (1.28-2.04)	1.74 (1.27-2.38)
35-45	0.95 (0.69-1.31)	1.40 (1.02-1.92)	2.34 (1.56-3.53)
Race/Ethnicity			
Non-Hispanic white	ref	ref	ref
Non-Hispanic Black	1.02 (0.75-1.40)	0.87 (0.66-1.15)	1.22 (0.85-1.76)
Hispanic	0.67 (0.50-0.91)	1.26 (0.98-1.63)	1.31 (0.96-1.78)
Non-Hispanic, other race	0.59 (0.39-0.90)	0.91 (0.66-1.27)	0.85 (0.56-1.27)
Tax Unit Income			
<400% FPL	1.50 (1.08-2.08)	1.98 (1.54-2.55)	1.19 (0.91-1.56)
≥400% of FPL	ref	ref	ref
Insurance			
Private	0.67 (0.52-0.87)	1.88 (1.49-2.36)	0.84 (0.64-1.11)
Public or Other	ref	ref	ref
Uninsured	4.40 (3.23-6.00)	5.18 (3.49-7.70)	1.52 (0.96-2.41)
Marital Status			
Married	0.89 (0.66-1.20)	0.86 (0.65-1.13)	0.81 (0.53-1.24)
Lives with partner	1.15 (0.82-1.62)	1.31 (0.93-1.83)	0.99 (0.61-1.60)
Widowed, separated, or divorced	1.30 (0.81-2.07)	1.08 (0.68-1.71)	1.05 (0.55-2.01)
Never married	ref	ref	ref
Family Size	0.97 (0.91-1.05)	0.99 (0.92-1.06)	0.97 (0.89-1.06)
Education			
Less than high school	1.11 (0.65-1.89)	1.69 (1.13-2.53)	0.89 (0.51-1.54)
High school diploma	1.17 (0.73-1.87)	1.57 (1.15-2.15)	1.08 (0.78-1.51)
Some college	1.08 (0.68-1.69)	1.21 (0.91-1.60)	1.02 (0.77-1.36)
College degree	ref	ref	ref
Employment Status			
Employed	1.05 (0.84-1.32)	1.31 (1.07-1.60)	0.97 (0.77-1.22)
Unemployed	ref	ref	ref
Self-Reported Health Status			
Poor	ref	ref	ref
Fair	0.62 (0.16-2.45)	0.55 (0.14-2.18)	0.38 (0.04-3.23)
Good	0.31 (0.08-1.18)	0.46 (0.12-1.75)	0.49 (0.06-3.84)
Very good	0.17 (0.05-0.65)	0.36 (0.10-1.38)	0.29 (0.04-2.24)
Excellent	0.12 (0.03-0.44)	0.23 (0.06-0.86(	0.24 (0.03-1.91)
Hospitalized in Past Year	0.89 (0.72-1.10)	1.13 (0.93-1.36)	1.15 (0.91-1.46)
Region			
Northeast	ref	ref	ref
Midwest	1.59 (1.07-2.38)	1.10 (0.81-1.50)	0.72 (0.52-1.00)
South	1.49 (1.03-2.17)	1.25 (0.93-1.68)	0.69 (0.50-0.94)
West	2.16 (1.45-3.22)	1.03 (0.76-1.38)	0.81 (0.58-1.15)

© 2021 Taylor K et al. *JAMA Network Open.*
